# The Core Role of Neutrophil–Lymphocyte Ratio to Predict All-Cause and Cardiovascular Mortality: A Research of the 2005–2014 National Health and Nutrition Examination Survey

**DOI:** 10.3389/fcvm.2022.847998

**Published:** 2022-05-12

**Authors:** Linguo Gu, Zhenkun Xia, Bei Qing, Hongzuo Chen, Wei Wang, Ying Chen, Yunchang Yuan

**Affiliations:** Department of Thoracic Surgery, The Second Xiangya Hospital of Central South University, Changsha, China

**Keywords:** NHANES, neutrophil-lymphocyte ratio, cardiovascular, mortality, all-cause

## Abstract

**Objective:**

To further supplement the previous research on the relationship between neutrophil–lymphocyte ratio (NLR) and all-cause and cardiovascular mortality, and construct clinical models to predict mortality.

**Methods:**

A total number of 2,827 observers were included from the National Health and Nutrition Examination Survey (NHANES) database in our research. NLR was calculated from complete blood count. According to the quartile of baseline NLR, those observers were divided into four groups. A multivariate weighted Cox regression model was used to analyze the association of NLR with mortality. We constructed simple clinical prognosis models by nomograms. Kaplan–Meier survival curves were used to depict cause-specific mortality. Restricted cubic spline regression was used to make explicit relationships between NLR and mortality.

**Results:**

This study recruited 2,827 subjects aged ≥ 18 years from 2005 to 2014. The average age of these observers was 51.55 ± 17.62, and 57.69% were male. NLR is still an independent predictor, adjusted for age, gender, race, drinking, smoking, dyslipidemia, and other laboratory covariates. The area under the receiver operating characteristic curves (AUCs) of NLR for predicting all-cause mortality and cardiovascular mortality were 0.632(95% CI [0599, 0.664]) and 0.653(95% CI [0.581, 0.725]), respectively, which were superior to C-reactive protein (AUCs: 0.609 and 0.533) and WBC (AUCs: 0.522 and 0.513). The calibration and discrimination of the nomograms were validated by calibration plots and concordance index (C-index), and the C-indexes (95% CIs) of nomograms for all-cause and cardiovascular mortality were 0.839[0.819,0.859] and 0.877[0.844,0.910], respectively. The restricted cubic spline showed a non-linear relationship between NLR and mortality. NLR > 2.053 might be a risk factor for mortality.

**Conclusion:**

There is a non-linear relationship between NLR and mortality. NLR is an independent factor related to mortality, and NLR > 2.053 will be a risk factor for prognosis. NLR and nomogram should be promoted to medical use for practicality and convenience.

## Introduction

Cardiovascular diseases or events (CVDs), such as acute myocardial infarction and coronary atherosclerotic heart disease, were once thought to be closely related to vascular endothelial damage, platelet aggregation, atherosclerotic plaque formation, and unstable plaque rupture (1, 2). Unstable shedding of plaque will block blood vessels and cause ischemic changes in organs. However, some new research believes that inflammation and oxidative stress also play a vital role in CVDs (3, 4). Inflammation is important pathogenesis of plaque formation, erosion of blood vessel walls, and plaque rupture (5, 6). Among all inflammatory cells, neutrophils and lymphocytes are the more active cells, participating in multiple processes of inflammatory response. Macrophages transform into foam cells by phagocytosing oxidized low-density lipoprotein (LDL) cholesterol and release inflammatory factors to recruit neutrophils to aggregate. Neutrophils participate in the inflammatory response in a variety of ways, such as releasing neutrophil elastase and proteinase-3, which corrode vessel walls and aggravate inflammatory response (7).

Neutrophil–lymphocyte ratio (NLR) is defined as the ratio of neutrophil count to lymphocyte count in peripheral blood. It is convenient to obtain the neutrophil count and lymphocyte count from complete blood count (CBC) data, and after a simple division calculation, the value of NLR can be obtained. More importantly, NLR has been confirmed to be closely related to CVDs, infectious and inflammatory diseases, tumors, and other diseases (8–11). In so many studies, we have noticed that the study of NLR both with cardiovascular and all-cause mortality is more valuable in clinical practice. Previous studies (12–14) also verified that NLR can reflect the degree of inflammation. However, how to put these studies into clinical medicine practice needs to be further explored.

The first aim of this research is to make it clearer whether there is a specific relationship between NLR and mortality. Second, we did this research in order to construct a practical prognosis prediction model and inform future research on this topic.

## Materials and Methods

### Study Population and Design

**FIGURE 1:**
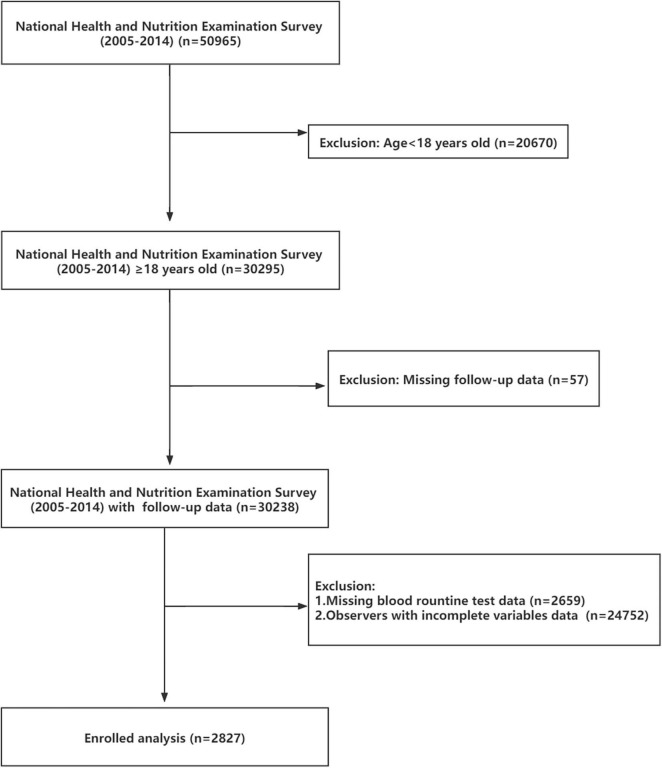
The research flowchart.

The National Health and Nutrition Examination Survey (NHANES^1^) database is public and free of charge. Beginning in the early 1960s, NHANES focused on a series of different population groups or health topics surveys. A complex, multistage, and nationwide probability sampling survey design was used each year when NHANES conducted the survey. NHANES does a cross-sectional study at each time point. Combined with interviews and physical examinations, NHANES provided great convenience for our study. NHANES approved research-related agreements and protocol, what is more, written informed consent was obtained from all participants. In our study, 50,965 respondents of all ages participated in the NHANES from 2005 to 2014. Focused on adult observers, we banned 20,670 participants from our study. Subsequently, 57 observers were excluded due to missing or incomplete follow-up data. After that, 2,659 records were deleted because CBC data showed no results. To reduce analytical errors, 24,752 participants were excluded because of incomplete variables data. Ultimately, 2,827 subjects with complete data were included (Figure 1).

### Assessment of Outcomes

We observe all-cause mortality and cardiovascular mortality as the end points. The mortality status of participants was authenticated by matching with the National Death Index.^2^ Referring to the International Classification of Disease, 10th Edition (ICD-10), Clinical Modification System codes, cardiovascular mortality was identified as I00–I09, I11, I13, I20–I51, or I60–I69 that was recorded in National Death Index (15). The above definition of cardiovascular mortality includes CVD and cerebrovascular disease both. We can also call cardiovascular mortality CVD mortality for short. All-cause mortality is defined as death from any cause.

### Covariate Data Obtained

We obtain our covariate data in the topic column of Questionnaire, Datasets, and Related Documentation from the NHANES database. We collected demographic data, laboratory data, examination data, and questionnaire data annually from 2005 to 2014. Baseline demographic data included age [we categorized into < 45, 45–60, > 60 years old, referred to literature (16)], gender (male or female), and race (Mexican American, Non-Hispanic White, Non-Hispanic Black, and other race). Body mass index [BMI; we categorized into < 25, 25–30, ≥ 30, referred to literature (17)] was obtained from examination data. Data on drinking (Yes or No), smoking (Yes or No), medical history of cancer (Yes or No), and recent physical condition (good, general, or poor, the situation at the time of the survey) were collected from a personal questionnaire. Hypertension was defined as meeting one of the following conditions: (a) the observers had ever been told by a doctor or other health professional that they had hypertension from questionnaire data; (b) the observers had been taking prescribed medicine for hypertension; and (c) three consecutive measurements of systolic blood pressure (SBP) > 140 mmHg or/and diastolic blood pressure (DBP) > 90 mmHg on different days. Diabetes was defined as meeting one of the following conditions (18): (a) a doctor or other health professional had informed the participants had diabetes or they had been taking pills for diabetes; (b) plasma fasting blood glucose (FBG) ≥ 7.0 mmol/L; and (c) glycated hemoglobin (GHB) (HbA1C) ≥ 6.5%. Participants will be considered for dyslipidemia if they meet one of the following criteria (19, 20): (a) doctors diagnosed them with dyslipidemia and/or they have been taking prescription medication; (b) serum triglycerides (TG) > 1.7mmol/L; (c) serum total cholesterol (TC) > 5.2mmol/L; and (d) serum LDL cholesterol > 3.4mmol/L and/or serum high-density lipoprotein (HDL) cholesterol < 1.0mmol/L. Other laboratory data, such as CBC data, standard biochemistry, serum lipids, and FBG, were performed under a unified standard. All blood specimen collection was conducted after overnight fasting.

### Statistical Analysis

SAS software (version 9.4)^3^ and R project (version 4.1.1)^4^ were used to perform data analysis; besides, the R project was also used for figure drawing. Observers were divided into four groups based on the quartile of baseline NLR levels [Q1: NLR < 1.54 (reference group), Q2: 1.55 ≤ NLR ≤ 2.04, Q3: 2.05 ≤ NLR ≤ 2.74, and Q4: NLR ≥ 2.75]. Continuous variables were checked for normal distribution before analysis, and these variables do not follow a normal distribution (Supplementary Table 1). Therefore, we used Kruskal–Wallis test, and data were presented as median (quantile 1st, quantile 3rd). For categorical variables, data were presented as numbers with proportions, and we used chi-squared tests for categorical variables. Cox regression analyses were used to investigate the hazard ratios (HRs) and 95% confidence intervals (CIs). NHANES database provided the weights of different samples; moreover, the database adopted a very complex survey design. Taking into account the above factors, we hereby used weighted Cox proportional hazards regression instead of traditional Cox regression to get a more reliable result with a less statistical error. The proportional hazards assumption of the Cox models was performed by R project, and our Cox model satisfied the assumption (Supplementary Figures 7A,B). Statistical indicators were presented as HR and 95% CIs. We also constructed three types of Cox regression models to explore the relationship between NLR and mortality. Kaplan–Meier survival analysis was used to explore the prognostic differences in different NLR groups. The log-rank test showed a statistical significance in subgroup differences. Nomograms were constructed to give a simple scoring system for the initial assessment of survival probability. Here we used stepwise regression and R project for graphing. We also used calibration plots and Harrell’s C-index to evaluate the nomograms, and four points were made in our calibration plots. Receiver operating characteristic (ROC) curves were used to compare the sensitivity and specificity of NLR, C-reactive protein (CRP), and white blood cell (WBC) count as clinical diagnostic indicators for predicting mortality. Last but not least, restricted cubic spline models were established to discover whether a linear relationship or not between NLR and HR. Once non-linear relationships were confirmed, we would make further explorations by using a three-piecewise linear regression model to illustrate the relationship between NLR and HR, and estimate the threshold point. In all those analyses, *p* for trend < 0.05 was considered statistically significant. To verify if the sample size for each group was reasonable, we visited the website^5^ and used sample size and power calculator. Accepting an alpha risk of 0.05 and a beta risk of 0.2 in a two-sided test, 664 subjects were required in the Q4 group and 1,918 in the Q1–Q3 groups to find a statistically significant difference between mortality rates. And our sample size was appropriate.

## Results

### Population Characteristics

A total of 2,827 adult observers were included in our study from the NHANES database (2005–2014). Among all these observers, the average age was 51.55 ± 17.62, and 57.69% were male. Basic characteristics were presented by quartile of baseline NLR level (Table 1).

**TABLE 1:** Demographic and clinic characteristics according to NLR levels.

	Total	NLR				P for trend
		Q1 < 1.54 (*n* = 738)	Q2 1.55–2.04 (*n* = 658)	Q3 2.05–2.74 (*n* = 705)	Q4 ≥ 2.75 (*n* = 726)	
Age (years)						< 0.001
<45	1033(36.54)	305(41.33)	259(39.36)	233(33.05)	236(32.50)	
45–64	1009(35.69)	289(39.16)	233(35.41)	268(38.01)	219(30.17)	
≥ 65	785(27.77)	144(19.51)	166(25.23)	204(28.94)	271(37.33)	
Gender (n, Male%)						< 0.001
Male	1631(57.69)	432(58.54)	356(54.10)	429(60.85)	414(57.02)	
Female	1196	306	302	276	312	
Race (n,%)						< 0.001
Mexican American	397(14.04)	114(15.45)	97(14.74)	112(15.89)	74(10.19)	
Other Hispanic	287(19.05)	86(11.65)	66(10.03)	72(10.21)	63(8.68)	
Non-Hispanic White	16874(59.2)	320(43.36)	395(60.03)	438(62.13)	521(71.76)	
Non-Hispanic Black	469(16.59)	218(29.54)	100(15.20)	83(11.77)	68(9.37)	
Drinking (n,%)						< 0.001
Yes	2361(83.52)	629(85.23)	546(82.98)	593(84.11)	593(81.68)	
No	466(16.48)	109(14.77)	112(17.02)	112(15.89)	133(18.32)	
Smoking (n,%)						< 0.001
Yes	1260(44.57)	330(44.72)	282(42.86)	320(45.39)	328(45.18)	
No	1567(55.43)	408(55.28)	376(57.14)	385(54.61)	398(54.82)	
Albumin(g/L)	42.00(40.00,44.00)	42.00(40.00,45.00)	42.00(40.00,44.00)	42.00(40.00,44.00)	42.00(39.00,44.00)	< 0.001*
HDL cholesterol (mmol/L)	1.29(1.09,1.69)	1.34(1.09,1.66)	1.29(1.06,1.58)	1.27(1.06,1.55)	1.32(1.09,1.60)	0.002*
BMI (kg/m^2^)						< 0.001
<25	881(31.16)	261(35.37)	198(30.09)	198(28.09)	224(30.85)	
25–29	948(33.53)	235(31.84)	223(33.89)	241(34.18)	249(34.30)	
≥ 30	998(35.30)	242(32.79)	237(36.02)	266(37.73)	253(34.85)	
WBC	6.90(5.70,8.40)	6.00(5.00,7.10)	6.60(5.60,7.80)	7.20(6.10,8.40)	8.10(6.60,9.90)	< 0.001*
CRP	0.22(0.09,0.52)	0.15(0.07,0.38)	0.20(0.09,0.44)	0.22(0.09,0.57)	0.33(0.12,0.76)	< 0.001*
Glycated hemoglobin (%)	5.50(5.20,5.80)	5.50(5.20,5.80)	5.50(5.20,5.80)	5.50(5.30,5.90)	5.50(5.20,5.90)	0.301*
Condition (n,%)						< 0.001
Poor	695(24.58)	179(24.25)	146(22.19)	183(25.96)	187(26.76)	
General	1166(41.25)	298(40.38)	272(41.34)	309(43.83)	287(39.53)	
Good	966(34.17)	261(35.37)	240(36.47)	213(30.21)	252(34.71)	
Cancer (n,%)						< 0.001
Yes	306(10.82)	52(7.05)	64(9.73)	81(11.49)	109(15.01)	
No	2521(89.18)	686(92.95)	594(90.27)	624(88.51)	617(84.99)	
Glucose(mmol/L)	5.61(5.16,6.16)	5.49(5.16,5.94)	5.61(5.22,6.16)	5.66(5.27,6.22)	5.66(5.16,6.33)	< 0.001*
Triglycerides (mmol/L)	1.31(0.94,1.91)	1.28(0.90,1.86)	1.33(0.96,1.94)	1.31(0.97,1.91)	1.32(0.93,1.92)	0.332*
Diabetes (n,%)						< 0.001
Yes	491(17.37)	113(15.31)	103(15.65)	132(18.72)	143(19.70)	
No	2336(82.63)	625(84.69)	555(84.35)	573(81.28)	583(80.30)	
Hypertension (n,%)						< 0.001
Yes	1225(43.33)	289(39.16)	250(37.99)	339(48.09)	347(47.80)	
No	1602(56.67)	449(60.84)	408(62.01)	366(51.91)	379(52.20)	
Dyslipidemia (n,%)						< 0.001
Yes	2052(72.59)	522(70.73)	496(75.38)	503(71.35)	531(75.34)	
No	775(27.41)	216(29.27)	162(24.62)	202(28.65)	195(26.86)	

*: Using Kruskal–Wallis test, data were presented as median(quantile 1st, quantile 3rd).

*Albumin, serum albumin level; WBC, white blood cell count; CRP: C-reactive protein. HDL: high-density lipoprotein, BMI: body mass index, Condition: recent physical condition, Cancer: with a medical history of cancer or not, Glucose: fasting blood glucose level.*

Obtaining higher NLR scores, subjects were most likely to be the aged (> 65 years old), male, drinkers, obese (BMI > 30 kg/m^2^), or individuals with dyslipidemia, higher WBC count, and higher CRP level (within the normal range). And they were less likely to be Non-Hispanic Black, poor recent physical condition persons, patients with cancer, and diabetics.

### The Relationship Between Neutrophil–Lymphocyte Ratio and Mortality

Three models were constructed to estimate NLR independent function and the association with mortality. Multivariate weighted Cox regression was used to evaluate the relationship between NLR and mortality after adjusting for potential confounders. HRs and 95% CIs are demonstrated in Table 2. Variables adjusted in Model 2 were consistent across all-cause and cardiovascular mortality subgroups, so were in Model 3.

**TABLE 2:** Multivariate Cox regression analysis of NLR with CVD and cause-specific mortality.

	NLR				P for trend
	Q_1_ < 1.54 HR (95%CI)	Q_2_ 1.55–2.04 HR (95%CI)	Q_3_ 2.05–2.74 HR (95%CI)	Q_4_ ≥ 2.75 HR (95%CI)	
Median follow-up time(years)	7.92	7.75	7.75	7.33	
CVD mortality					
Death, n (%)	10(1.36)	10(1.52)	16(2.27)	32(4.41)	
Death/person-years	45/5688	28/5020	70/5287	137/5222	
Unadjusted Model 1	1[Reference]	2.04(0.73,5.68)	2.04(0.52,8.08)	5.91(2.52,13.87)	< 0.001
Model 2	1[Reference]	2.28(0.84,6.19)	1.37(0.37,5.12)	3.53(1.44,8.65)	< 0.001
Model 3	1[Reference]	2.42(0.86,6.83)	1.50(0.40,5.68)	3.19(1.35,7.55)	< 0.001
All-cause mortality					
Death, n (%)	65(8.81)	66(10.03)	80(11.35)	162(22.31)	
Death/person-years	295/5688	315/5020	333/5287	688/5222	
Unadjusted Model 1	1[Reference]	1.13(0.78,1.65)	1.14(0.78,1.68)	2.59(1.80,3.72)	< 0.001
Model 2	1[Reference]	1.14(0.80,1.63)	0.78(0.54,1.12)	1.59(1.09,2.32)	< 0.001
Model 3	1[Reference]	1.17(0.80,1.72)	0.79(0.54,1.17)	1.44(0.97,2.14)	< 0.001

*1. Percentages and mortality rates were estimated using United States population weights.*

*2. Values are numerical values or weighted hazard ratios (95% CIs).*

*Model 2: adjusted for Age + Gender + Race + Drinking + Smoking + Condition + Albumin.*

*Model 3: Model 2 + BMI + Cancer + WBC + HDL + Glycated hemoglobin + Triglycerides + CRP + Dyslipidemia + Glucose + Hypertension + Diabetes.*

#### All-Cause Mortality

In Mode 1, variables were unadjusted. The higher the NLR level was, the more evidently risk of all-cause mortality increased (Q4 vs. Q1: HR 2.59, 95% CI [1.80, 3.72]). Per one increment of NLR was equal to increasing HR of Q2, Q3, and Q4 group by 13%, 14%, and 159%, respectively, compared with Q1 group. In Model 2, we made an adjustment for age, gender, race, drinking, smoking, recent physical condition, and albumin level. The variables of MBI, HDL, WBC, cancer, hypertension, diabetes, GHB level, triglycerides, CRP, dyslipidemia, and FBG level were also adjusted in Model 3, in addition to the variables in Model 2. It was still statistically significant between NLR levels and all-cause mortality after multivariable adjustment.

**FIGURE 2:**
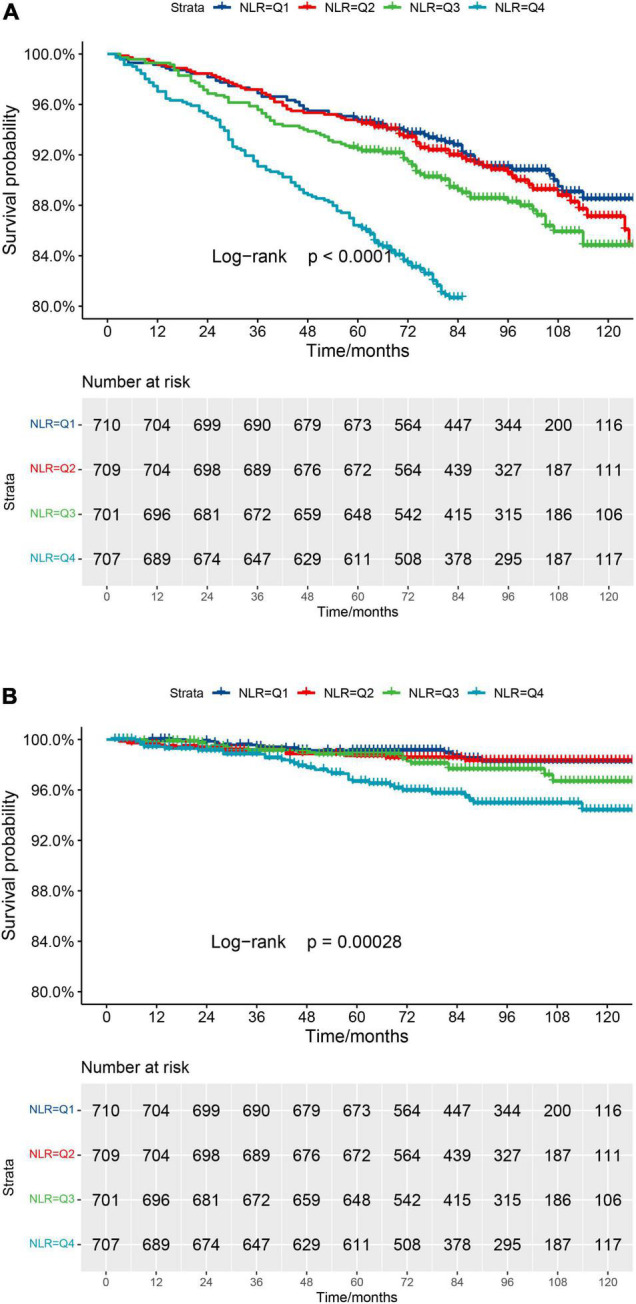
**(A)** Kaplan–Meier survival curve for all-cause mortality. **(B)** Kaplan–Meier survival curve for cardiovascular mortality.

Kaplan–Meier curve (Figure 2A) confirms the above conclusion. Compared with lower NLR levels, observers with higher NLR scores get a steeper decline in survival over a 10-year follow-up (log-rank *p* for trend < 0.0001).

#### Cardiovascular Mortality

In Model 1, no variables were adjusted. Higher NLR levels are associated with a higher risk of cardiovascular mortality (Q4 vs. Q1: HR 5.91, 95% CI [2.52, 13.87]). A similar conclusion was found in adjusted Models 2 and 3. The above results indicate that NLR is an independent risk factor, and NLR increment within a certain range will increase the risk of cardiovascular mortality (*p* < 0.001).

In order to make the above results more clear, the Kaplan–Meier curve (Figure 2B) was constructed. The highest risk is observed in the group with the highest NLR level (Group Q4, log-rank *p* for trend = 0.00028). However, it seems not statistically significant in Group Q1, compared to Group Q2.

### Survival Prediction and Evaluation

To construct a simple clinical scoring system for predicting mortality, nomograms were considered. Related parameters of each variable in the nomograms are shown in Table 3.

**TABLE 3:** Parameters of Cox regression model in nomogram for all-cause and cardiovascular mortality.

	All-cause mortality			Cardiovascular mortality		
	Coef	HR	*p* for trend	Coef	HR	*p* for trend
Age	0.0775	1.0806	< 0.0001	0.0770	1.0800	< 0.0001
Gender	0.4117	1.5094	0.0004	0.9652	2.6252	0.0022
Race	0.1223	1.1301	0.0697	—	—	—
Smoking	0.4535	1.5738	0.0003	—	—	—
Condition	-0.3937	0.6746	< 0.0001	-0.3901	0.6770	0.0184
Albumin	-0.0868	0.9169	< 0.0001	-0.0884	0.9154	0.0164
LDL cholesterol	-0.2313	0.7935	< 0.0001	-0.3204	0.7259	0.0278
Glucose	0.0485	1.0497	0.0451	—	—	—
BMI	-0.0300	0.9704	0.0020	–0.0596	0.9422	0.0171
NLR	0.1692	1.1843	< 0.0001	0.1975	1.2184	< 0.0001
GHB	—	—	—	0.3322	1.3940	0.0067
Cancer	—	—	—	–0.9060	0.4041	0.0187
Hypertension	—	—	—	0.6027	1.8271	0.0479
Diabetes	—	—	—	–0.9346	0.3928	0.0235
C-index: 0.839(0.819,0.859)				C-index: 0.877(0.844,0.910)		

*Coef: regression coefficient; HR: hazard ratio, C-index: concordance index (presented as value and 95%CI), Condition: recent physical condition, Albumin: serum albumin level, LDL: low-density lipoprotein, Glucose: fasting blood glucose, BMI: body mass index, NLR: neutrophil–lymphocyte ratio, GHB: glycated hemoglobin.*

**FIGURE 3:**
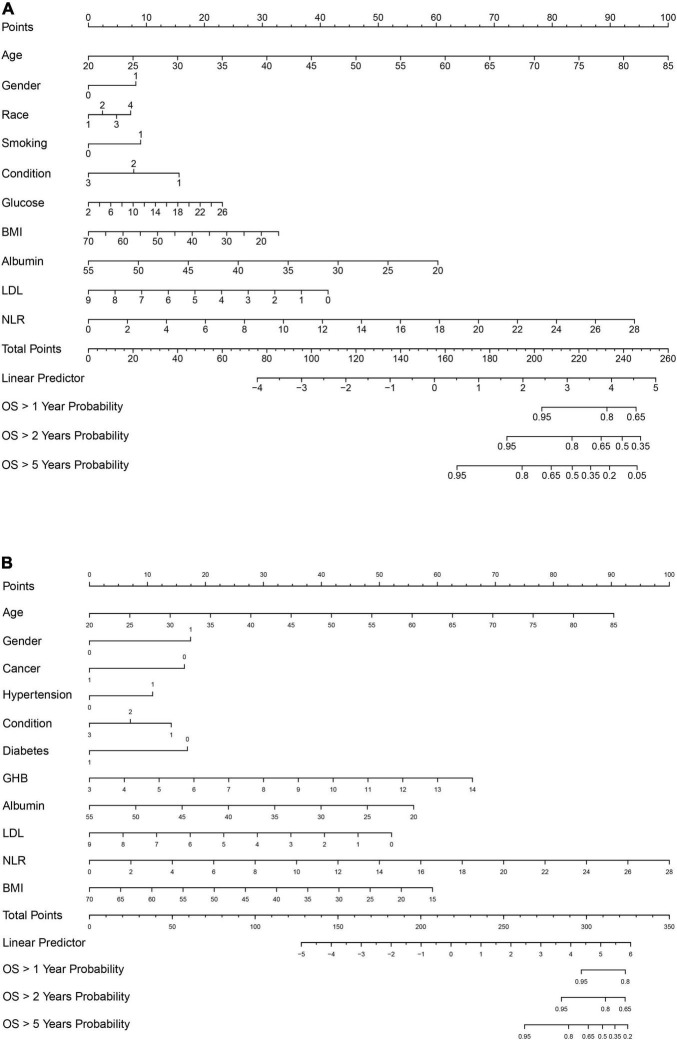
**(A)** Nomogram model for all-cause mortality. **(B)** Nomogram model for cardiovascular mortality. Race: 1, Mexican American; 2, other race; 3, non-Hispanic White; 4, non-Hispanic Black. Gender: 1, male; 0, female. For hypertension, diabetes, smoking, and cancer: 1, Yes; 0, No. Condition: recent physical condition, and for Condition: 1, poor; 2, general; 3, good. Glucose, fasting blood glucose. BMI, body mass index. Albumin, serum albumin level; LDL; low-density lipoprotein cholesterol level; GHB, glycated hemoglobin; OS, overall survival.

For all-cause mortality (Figure 3A), age, gender, race, smoking, recent physical conditions, plasma FBG level, BMI, LDL, serum albumin level, and NLR were used to construct a nomogram. For cardiovascular mortality (Figure 3B), variables of race, smoking, and plasma FBG level were replaced by hypertension, diabetes, and GHB level. Besides, we added the variable “cancer” to the nomogram. A 65-year old non-Hispanic Black man, with a history of tobacco use, complains of recent poor health. His laboratory test results are as followed: FBG: 8.0 mmol/L, LDL: 2.0 mmol/L, albumin: 30 g/L, BMI: 25 kg/m^2^, and NLR: 6.0. Nomogram calculations for all-cause mortality are as follows:

Age = 65 years old, which corresponds to 68 points;

Gender = male, which corresponds to 8 points;

Race = non-Hispanic Black, which corresponds to 7 points;

…

NLR = 6.0, which corresponds to 20 points.

The total points ≈68 + 8 + 7 + … + 20 ≈ 235, which corresponds to 1-year survival [OS (overall survival) > 1 year probability] of 78%, 2-year survival (OS > 2 years probability) of 60%, and 5-year survival (OS > 5 years probability) of 20%. This calculation method is also applicable to the nomogram calculations of cardiovascular mortality. C-index and calibration plots (Supplementary Figures 4A–F) were performed to evaluate the discrimination and calibration of the nomograms. Our C-indexes (95% CIs) of nomograms for all-cause and cardiovascular mortality were 0.839[0.819, 0.859] and 0.877[0.844, 0.910], respectively. The above results suggest that our nomograms were discriminative and calibrated.

### Neutrophil–Lymphocyte Ratio Performed a Superior Role in Mortality Prediction

**FIGURE 4:**
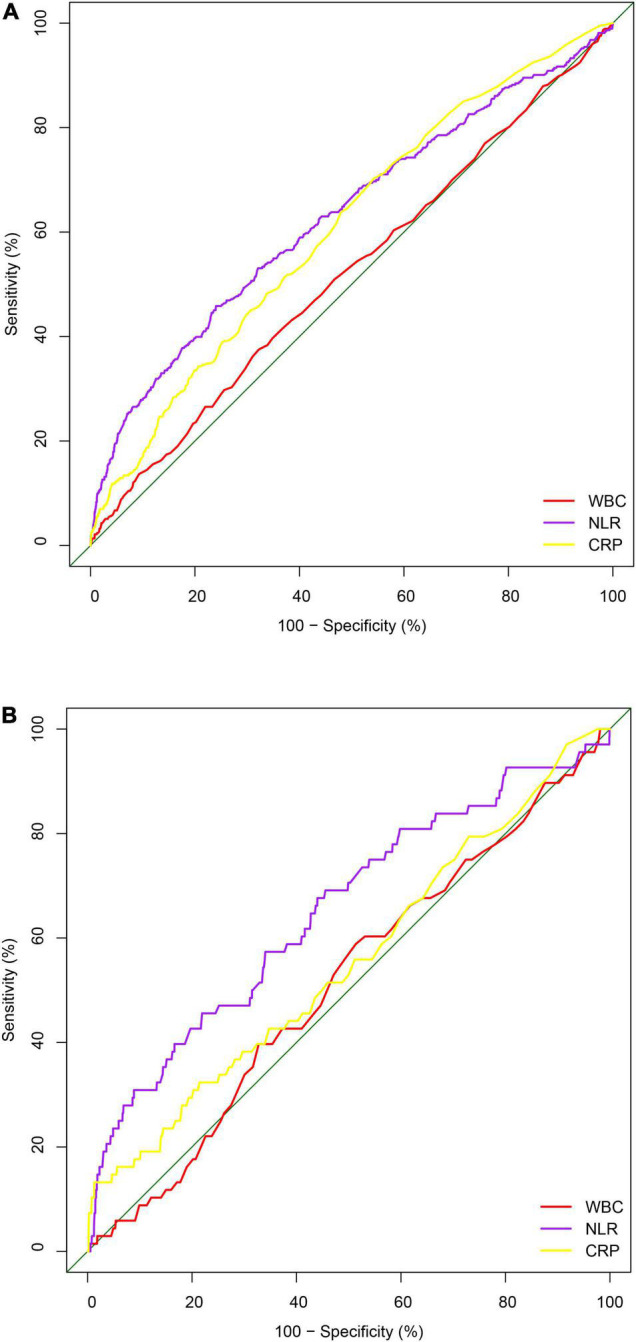
**(A)** ROC curve analysis of NLR, compared to CRP and WBC to predict all-cause mortality. **(B)** ROC curve analysis of NLR, compared to CRP and WBC to predict cardiovascular mortality. CRP, C-reactive protein (mg/dl); WBC, white blood cell count in CBC (× 10^9^/L).

To verify whether NLR improved predictive mortality ability than classical inflammatory markers (WBC count and CRP), ROC curves (Figures 4A,B) were established. The area under the ROC curves (AUCs) of NLR for all-cause and cardiovascular mortality were 0.632(95% CI [0599, 0.664]) and 0.653(95% CI [0.581, 0.725]), respectively. The AUC of NLR is the largest, which means the predictive ability of NLR is superior to CRP (AUCs: 0.609 and 0.553) and WBC (AUCs: 0.522 and 0.513).

### Non-linear Relationships Between Neutrophil–Lymphocyte Ratio and Mortality

**FIGURE 5:**
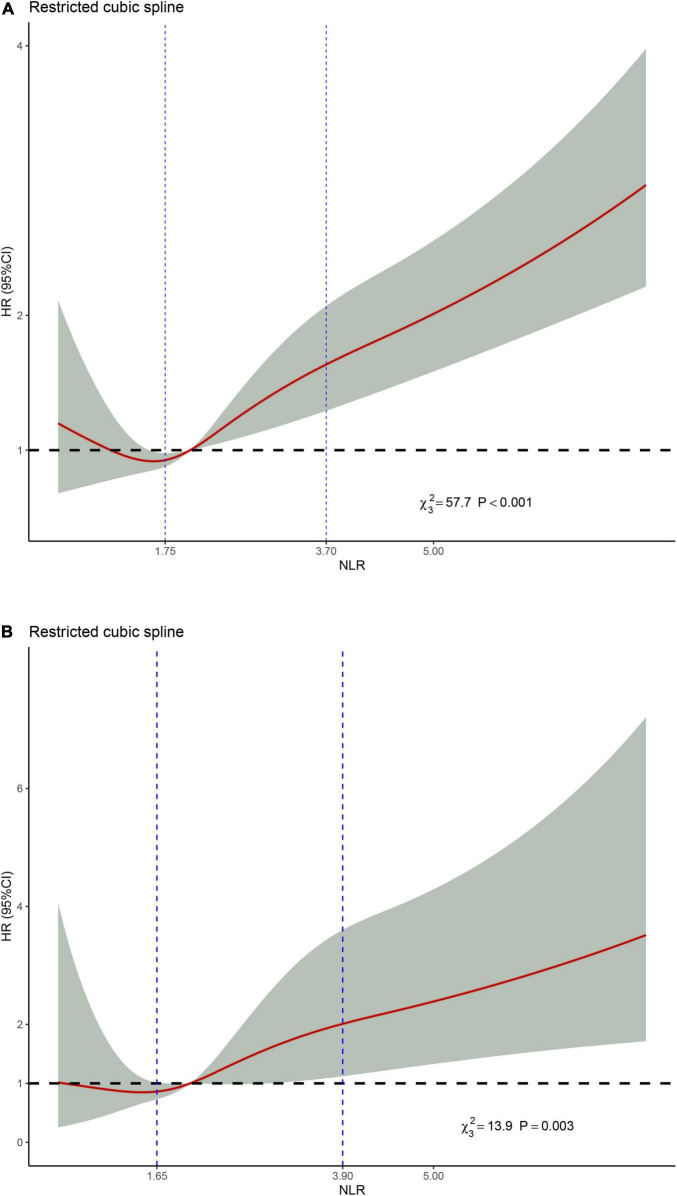
**(A)** Non-linear relationship between NLR and all-cause mortality. Adjusted for age, gender, race, smoking, condition, glucose, BMI, and albumin. **(B)** Non-linear relationship between NLR and cardiovascular mortality. Adjusted for age, gender, cancer, hypertension, condition, diabetes, BMI, glycated hemoglobin, and albumin.

Restricted cubic spline regression was used to make explicit relationships between NLR and HR. Considering a non-linear relationship between NLR and HR, three-piecewise linear regression was determined to be the most suitable model (non-linear *p* < 0.001 and non-linear *p* = 0.003, respectively). For all-cause mortality (Figure 5A), we adjusted for age, gender, race, smoking, recent physical condition, FBG, BMI and albumin. As the NLR increased, the HR value showed a decreasing trend when NLR < 1.75. HR increased significantly when 1.75 < NLR < 3.70 (the second stage). When NLR > 3.70, the HR value continued to show an increasing trend but was not as obvious as the second stage. For cardiovascular mortality (Figure 5B), age, gender, cancer, hypertension, recent physical condition, diabetes, BMI, GHB, and albumin were adjusted. HR decreased up to a valley at the NLR level of 1.65 and then increased with a higher NLR level. HR got a steeper ascension during the second stage both in all-cause and cardiovascular mortality. A clinically meaningful threshold point of NLR level is 2.053, where HR equals to 1.0.

## Discussion

This was a large-scale and multiethnic cross-sectional analysis, which exposed the correlation between NLR scores and all-cause and cardiovascular mortality in American adults. NLR was independently associated with all-cause mortality and cardiovascular mortality based on the above results. After the adjustment of the variable, the above conclusion still holds. Unlike the previous study on this topic (21–24), our research further clarified the exact relationship between NLR and two types of mortality, and we also identified the valuable threshold points. Besides, we constructed a prognostic-related nomogram model for the first time.

Produced by the bone marrow, neutrophils and lymphocytes are the principal members involved in inflammation of the leukocyte family. Unlike lymphocytes, neutrophils are more active in inflammation because of their chemotaxis. Once neutrophil surface receptors bind to chemokines, neutrophils deform and migrate to where inflammation occurs (25). After that, neutrophils will release cytokines, which also affect the activation of lymphocytes, and participate in inflammation. Inflammatory cytokines released by neutrophils accelerate the progression of CVDs (24). Therefore, many CVDs, such as coronary atherosclerotic heart disease and acute myocardial infarction, are considered to be the result of inflammation (26). Some anti-inflammatory and anti-cytokine therapies have been studied to treat coronary heart disease (27–29). In healthy individuals, the NLR value is approximately 1.65–1.70 (9). Our result was 2.303 ± 1.278 in all 2,827 observers, higher than the literature reported. *In vitro* experiments in mice confirmed that the proportion of neutrophils increased significantly after myocardial infarction (30). Stephen et al. have told us that decreased lymphocyte count resulted in a poor prognosis of CVDs (31). Besides, we speculate that during inflammation, our body needs more neutrophils rather than lymphocytes, and the bone marrow tends to produce neutrophils. Thence, the increased neutrophils and the reduction in lymphocytes will lead to a poor prognosis of CVDs. When neutrophils increase and lymphocytes decrease, the proportion of the two will increase. That is one of the reasons why an increase in the NLR level will lead to a poor prognosis.

There are many commonly used inflammation indexes, such as CRP, erythrocyte sedimentation rate, procalcitonin, and WBC count. In all these indexes, CRP and high-sensitivity CRP are the most studied (32). Regardless of the sensitivity and specificity of these indicators, the cost of measuring these indicators is not cheap in clinical practice. However, the NLR is relatively easy to obtain, and the cost of blood routine tests is very economical. Our ROC curves also confirmed that the sensitivity and specificity of NLR are higher than those of CRP and WBC. There are many studies on the impact of NLR on CVDs or cardiovascular mortality (14, 33–35). However, there is no simple and convenient model to predict survival, yet. In order to get a convenient prediction model, we constructed nomograms using stepwise regression analysis with the help of the R project. Nomogram used to be a simple graphical representation of a statistical predictive model and was widely used for cancer prognosis (36, 37). A good nomogram will allow the doctor to get some useful information more quickly. Two primary ways of nomograms are used in clinical medicine, one is what we show in this paper, and the other is a computer-based calculator, where specific variables are entered and the likelihood of an event is computed (37, 38). To answer a focused clinical question, a nomogram must be constructed carefully. C-index and calibration plots are used to appraise nomograms, and our nomograms are excellent.

Many factors can affect NLR levels, such as dietary nutritional factors, pharmacological treatments, and comorbidities (39–41). Comorbidities, such as chronic obstructive pulmonary disease (COPD), have an influence on the NLR level. Relative lymphopenia and further increment of NLR value have been demonstrated to be a risk factor in elderly patients with severe COPD (40, 42, 43). Regrettably, data on comorbidities of observers are missing from the database. More research is required to supplement this topic.

There were three strengths in our research. First, we have an adequate sample size of 2,827 observers with complete data. Second, we are the first to construct nomogram models. Finally, we used the R project and weighted Cox regression analysis instead of classical Cox regression to get a credible result. In addition, our research was limited in certain respects. First, the retrospective nature of this paper is a further limitation; we cannot get dynamic variable data. Up to now, the follow-up outcome data published by The National Center for Health Statistics are only up to 2014, and we cannot obtain follow-up data after 2014. Second, to the year 2014, fewer observers died, which will lead to statistical errors. We suspect lower AUCs and wider 95% CIs in cardiovascular mortality for this reason. Last, nomogram performance lacks accepted reporting standards and varies widely. More prospective studies are required to optimize the nomograms and our research.

## Conclusion

In conclusion, our research suggests that NLR is independently associated with all-cause and cardiovascular mortality, and NLR > 2.053 may be a risk factor of mortality. The NLR value is a valuable index and should be recommended for use.

## Data Availability Statement

The datasets presented in this study can be found in online repositories. The names of the repository/repositories and accession number(s) can be found in the article/Supplementary Material.

## Ethics Statement

Ethical review and approval was not required for the study on human participants in accordance with the local legislation and institutional requirements. The patients/participants provided their written informed consent to participate in this study.

## Author Contributions

YY conceived and designed this study. LG analyzed all data and drafted this manuscript. ZX, BQ, and HC collected all data. WW and YC consulted related reference literature. All authors read and approved the final manuscript.

## Conflict of Interest

The authors declare that the research was conducted in the absence of any commercial or financial relationships that could be construed as a potential conflict of interest.

## Publisher’s Note

All claims expressed in this article are solely those of the authors and do not necessarily represent those of their affiliated organizations, or those of the publisher, the editors and the reviewers. Any product that may be evaluated in this article, or claim that may be made by its manufacturer, is not guaranteed or endorsed by the publisher.

## Footnotes

1
https://www.cdc.gov/nchs/nhanes/

2
https://ftp.cdc.gov/pub/Health_Statistics/NCHS/datalinkage/linked_mortality

3
https://www.sas.com/en_us/home.html

4
https://www.rproject.org/

5
https://www.imim.es/ofertadeserveis/software-public/granmo/

## Abbreviations

NLR: Neutrophil–lymphocyte ratio
NHANES: National Health and Nutrition Examination Survey
CBC: Complete blood count
ROC: Receiver operating characteristic
LDL: Low-density lipoprotein
HDL: High-density lipoprotein
ICD: International Classification of Disease
CVD: Cardiovascular disease or event
BMI: Body mass index
GHB: Glycated hemoglobin
CI: Confidence interval
HR: Hazard ratio
C-index: Concordance index
CRP: C-Reactive protein
Coef: Regression coefficient
COPD: Chronic obstructive pulmonary disease.
