# Supplementary material for: The Core Role of Neutrophil–Lymphocyte Ratio to Predict All-Cause and Cardiovascular Mortality: A Research of the 2005–2014 National Health and Nutrition Examination Survey
Source: Front Cardiovasc Med. 2022 May 12;9:847998. doi: 10.3389/fcvm.2022.847998 (PMC9133381; doi:10.3389/fcvm.2022.847998)
Supplement: Supplementary file 1 [file Table_1.DOCX]

Table4 The result of normal distribution test for continuous variables

|  | Q1 | | Q2 | | Q3 | | Q4 | |
| --- | --- | --- | --- | --- | --- | --- | --- | --- |
|  | D | p | D | p | D | p | D | p |
| Albumin | 0.081, Pr > D | <0.01 | 0.091, Pr > D | <0.01 | 0.096, Pr > D | <0.01 | 0.116, Pr > D | <0.01 |
| HDL | 0.088, Pr > D | <0.01 | 0.090, Pr > D | <0.01 | 0.117, Pr > D | <0.01 | 0.086, Pr > D | <0.01 |
| WBC | 0.086, Pr > D | <0.01 | 0.073, Pr > D | <0.01 | 0.070, Pr > D | <0.01 | 0.066, Pr > D | <0.01 |
| CRP | 0.226, Pr > D | <0.01 | 0.253, Pr > D | <0.01 | 0.244, Pr > D | <0.01 | 0.317, Pr > D | <0.01 |
| GHB | 0.211, Pr > D | <0.01 | 0.225, Pr > D | <0.01 | 0.219  ,Pr > D | <0.01 | 0.195, Pr > D | <0.01 |
| Glucose | 0.229, Pr > D | <0.01 | 0.238, Pr > D | <0.01 | 0.225, Pr > D | <0.01 | 0.203, Pr > D | <0.01 |
| Triglycerides | 0.113, Pr > D | <0.01 | 0.113, Pr > D | <0.01 | 0.109, Pr > D | <0.01 | 0.109, Pr > D | <0.01 |

D: Kolmogorov-Smirnov test.

All p value<0.05, indicating that the variables do not follow a normal distribution.

**Figure legends in supplementary materials**

Figure4A, Fifure4B, and Figure4C: Calibration plots for nomogram of all-cause mortality. Figure4A: calibration for OS>1 year probability, Figure4B: calibration for OS>2 years probability, Figure4C: calibration for OS>5 years probability.

Figure4D, Figure4E, and Figure4F: Calibration plots for nomogram of cardiovascular mortality. Figure4A: calibration for OS>1 year probability, Figure4B: calibration for OS>2 years probability, Figure4C: calibration for OS>5 years probability.

Figure7A and Figure7B: R project code for the proportional hazards assumption of the Cox models. All p value >0.05, indicating that the Cox model satisfied the assumption.
