# Supplementary figures and images for: The Core Role of Neutrophil–Lymphocyte Ratio to Predict All-Cause and Cardiovascular Mortality: A Research of the 2005–2014 National Health and Nutrition Examination Survey
Source: Front Cardiovasc Med. 2022 May 12;9:847998. doi: 10.3389/fcvm.2022.847998 (PMC9133381; doi:10.3389/fcvm.2022.847998)

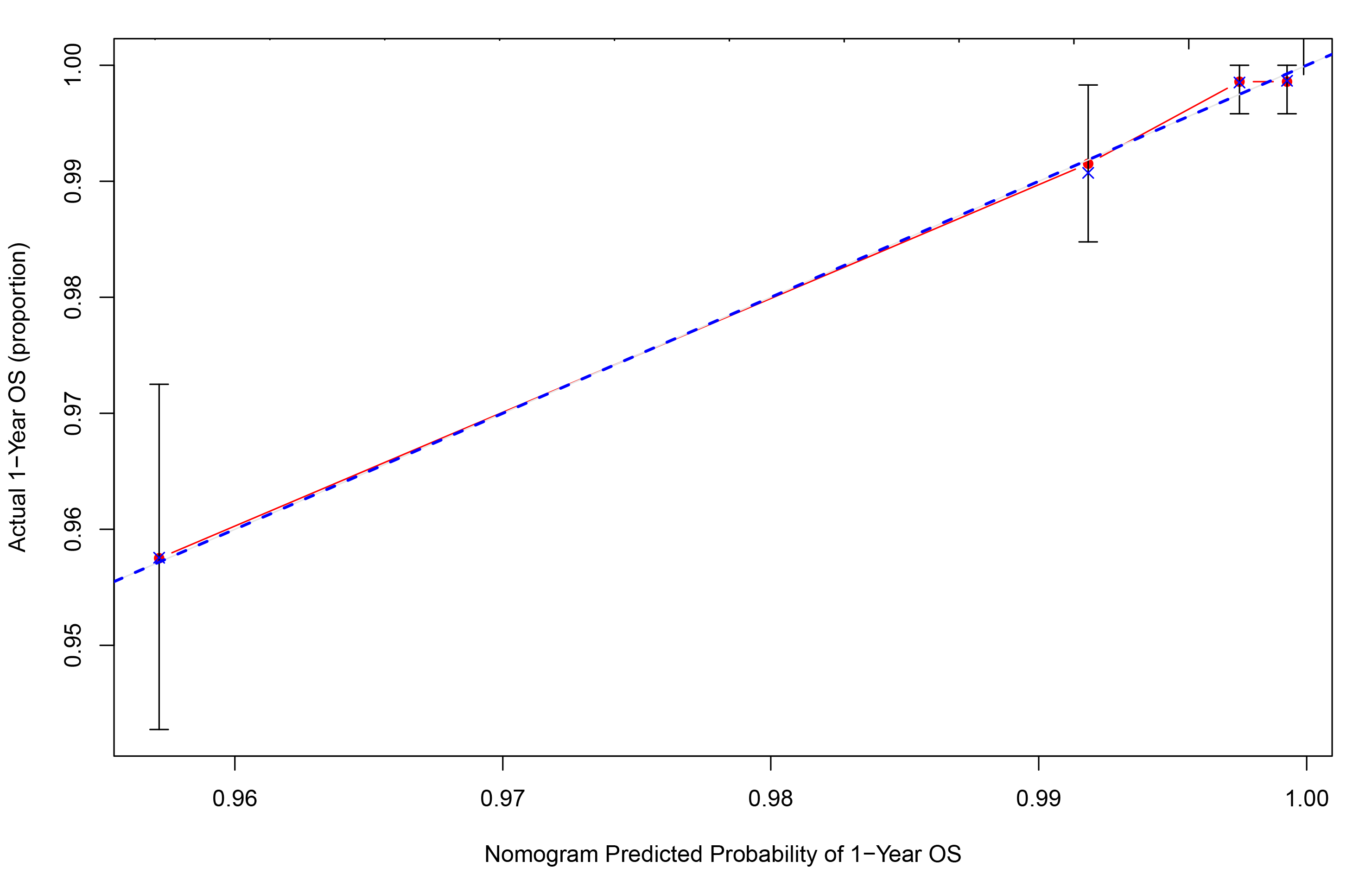

Supplement: Supplementary file 2 [file Image_1.TIF]

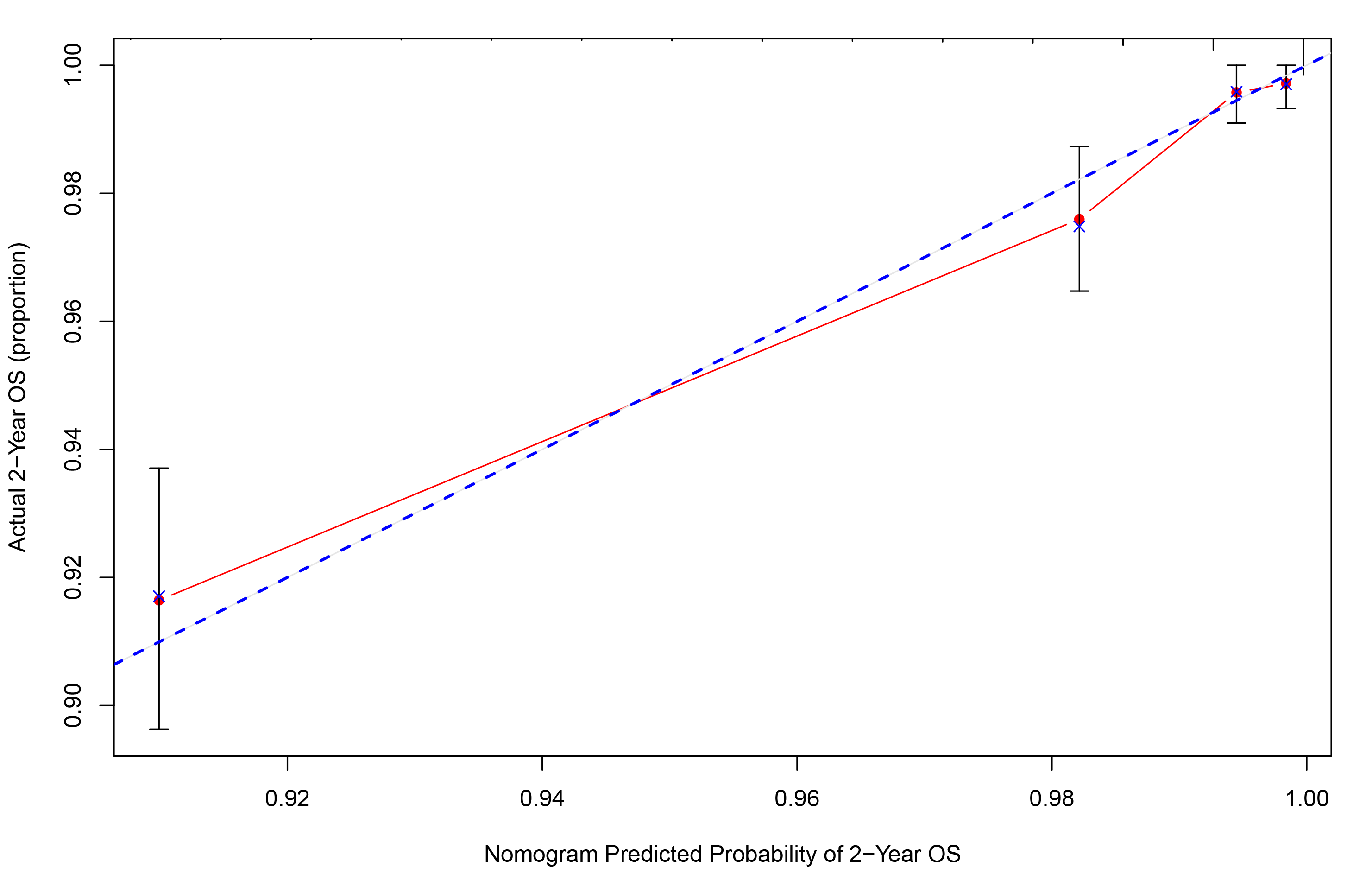

Supplement: Supplementary file 3 [file Image_2.TIF]

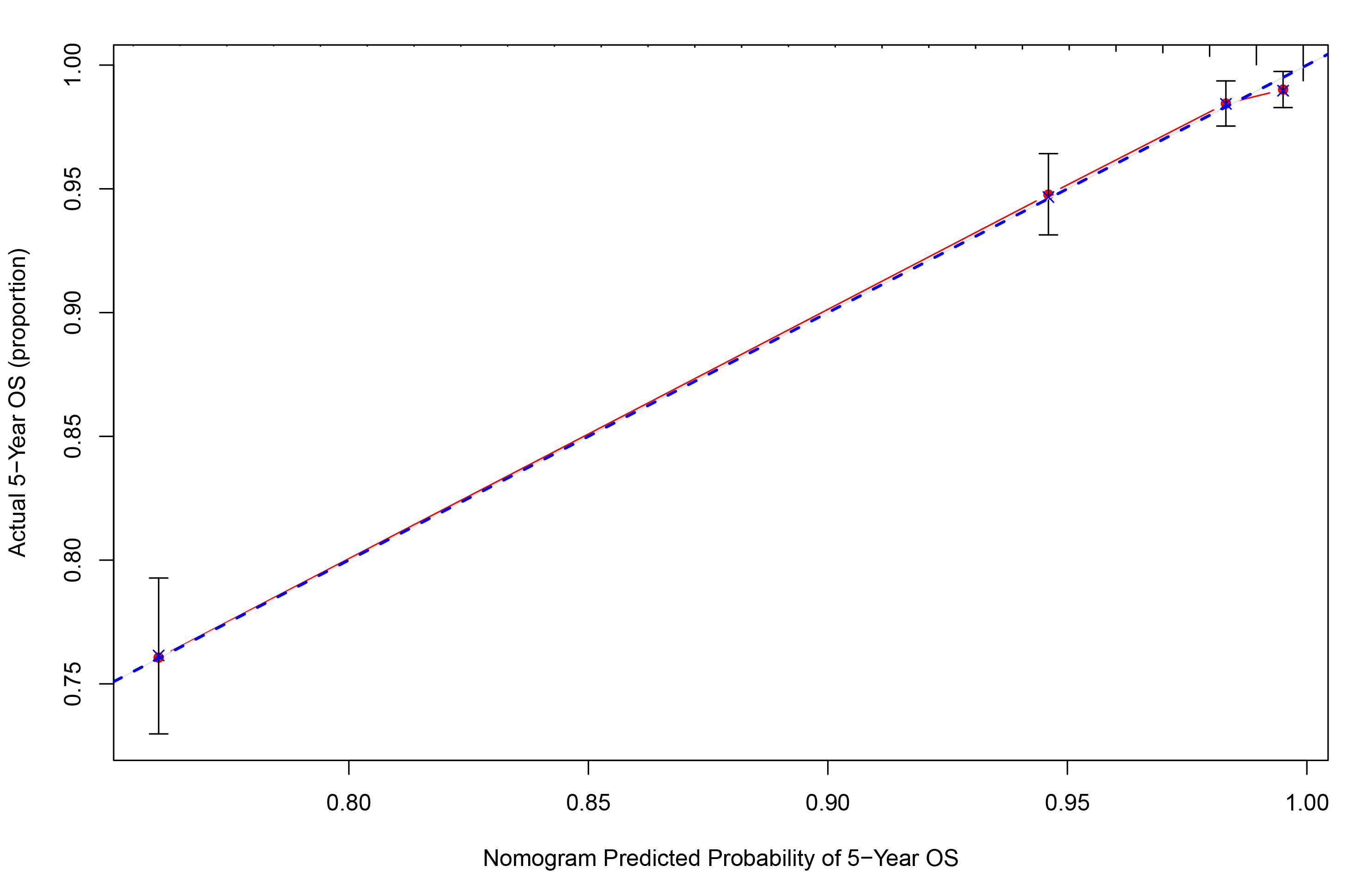

Supplement: Supplementary file 4 [file Image_3.TIF]

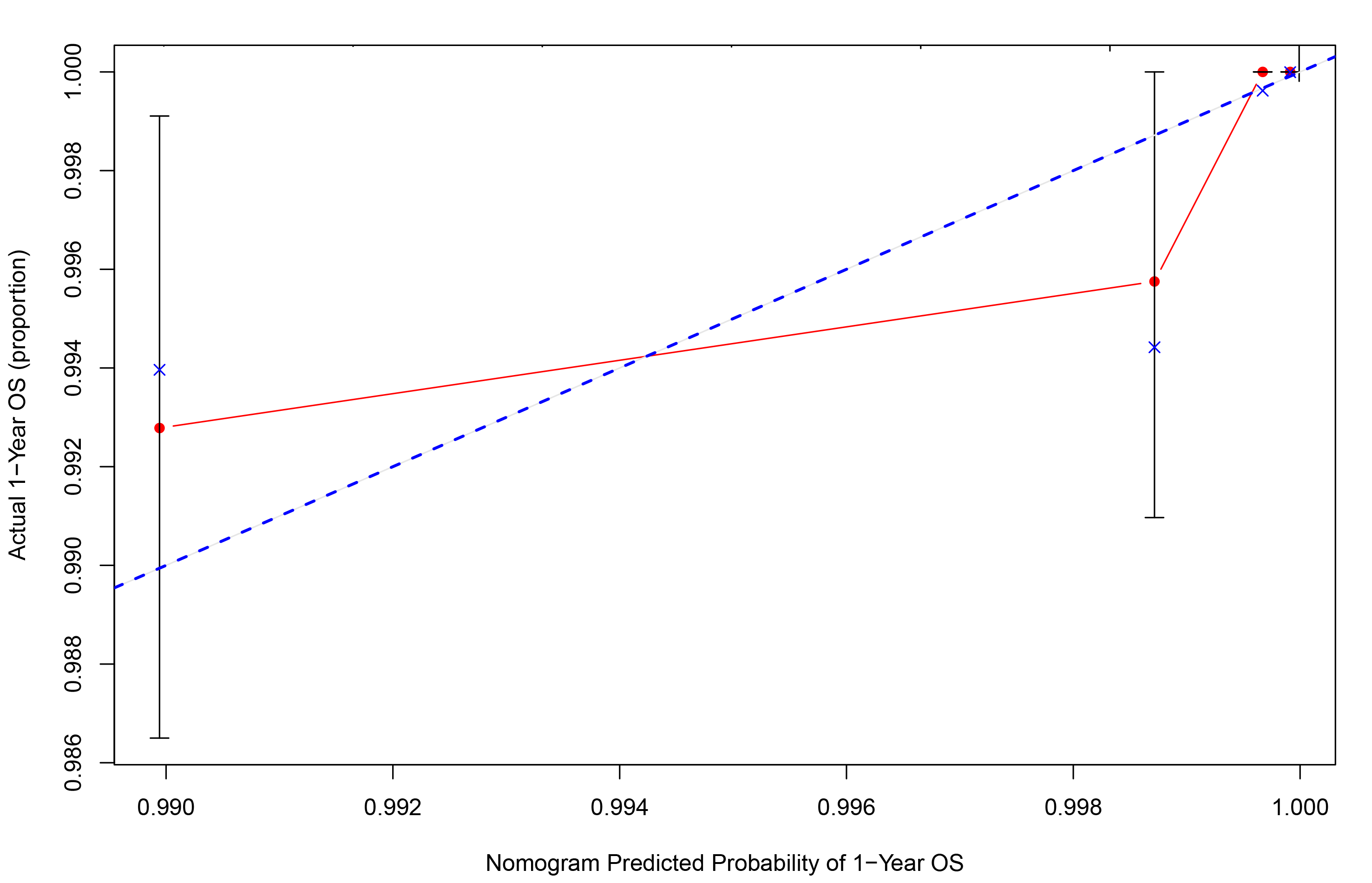

Supplement: Supplementary file 5 [file Image_4.TIF]

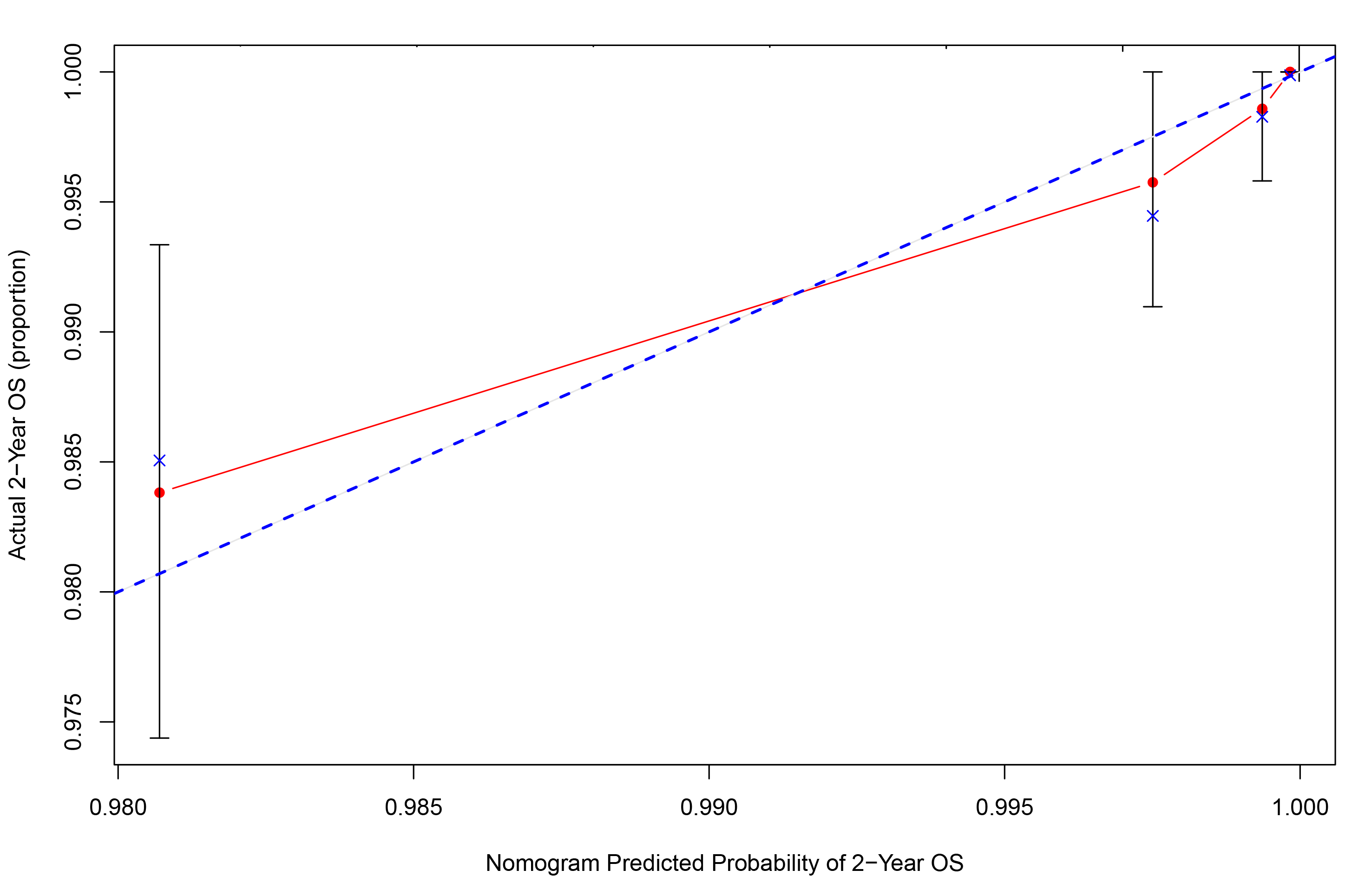

Supplement: Supplementary file 6 [file Image_5.TIF]

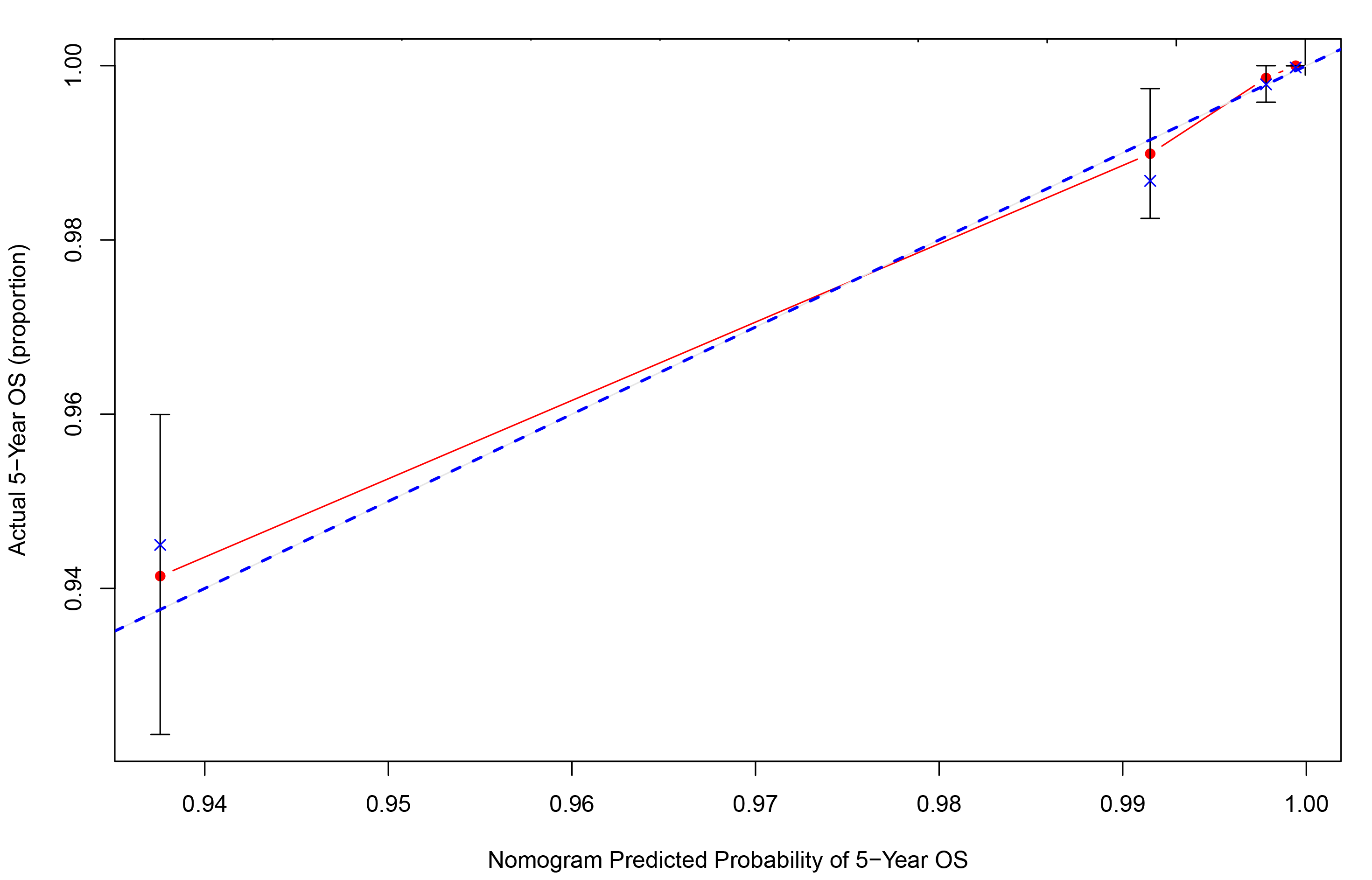

Supplement: Supplementary file 7 [file Image_6.TIF]

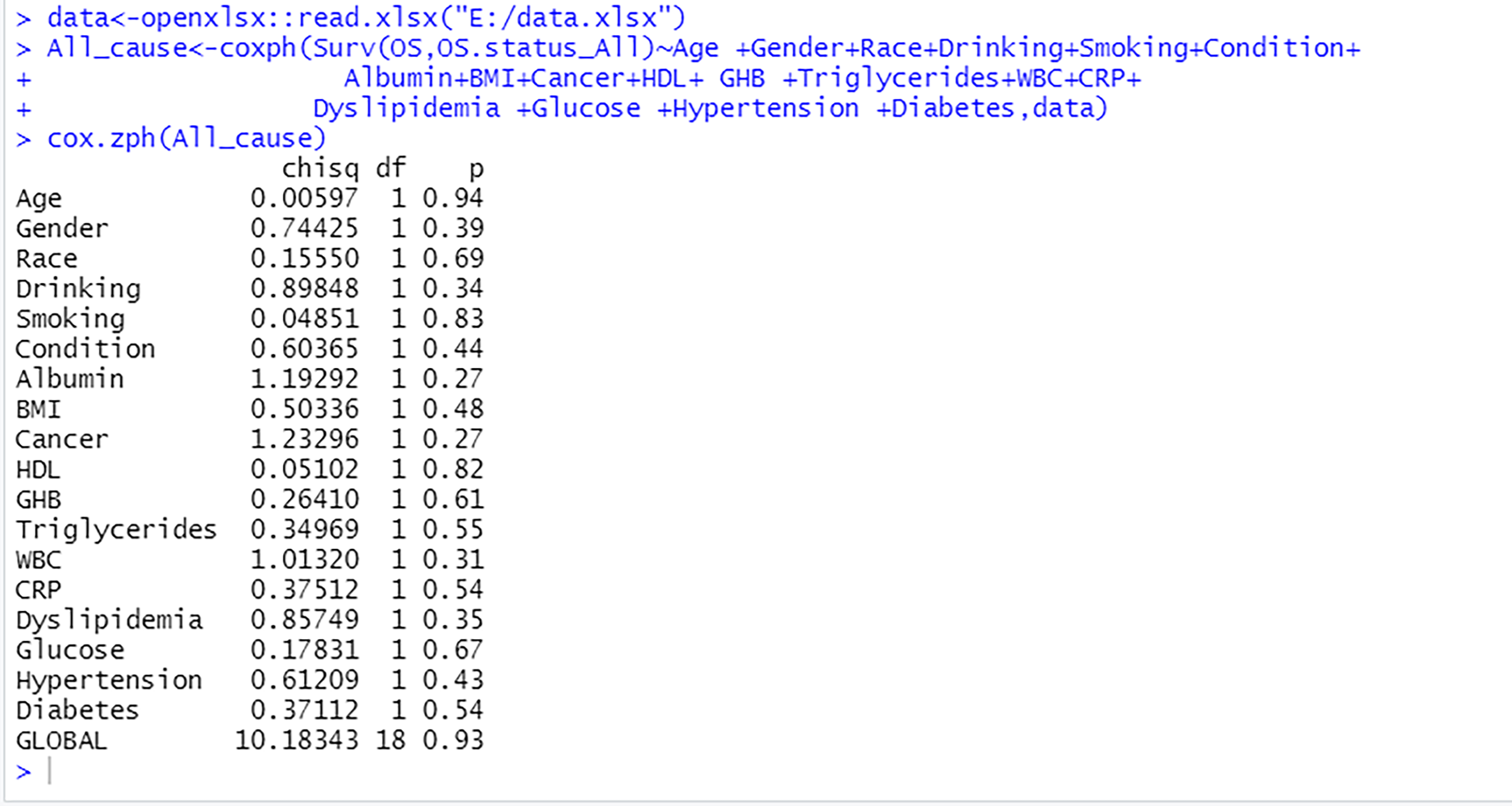

Supplement: Supplementary file 8 [file Image_7.TIF]

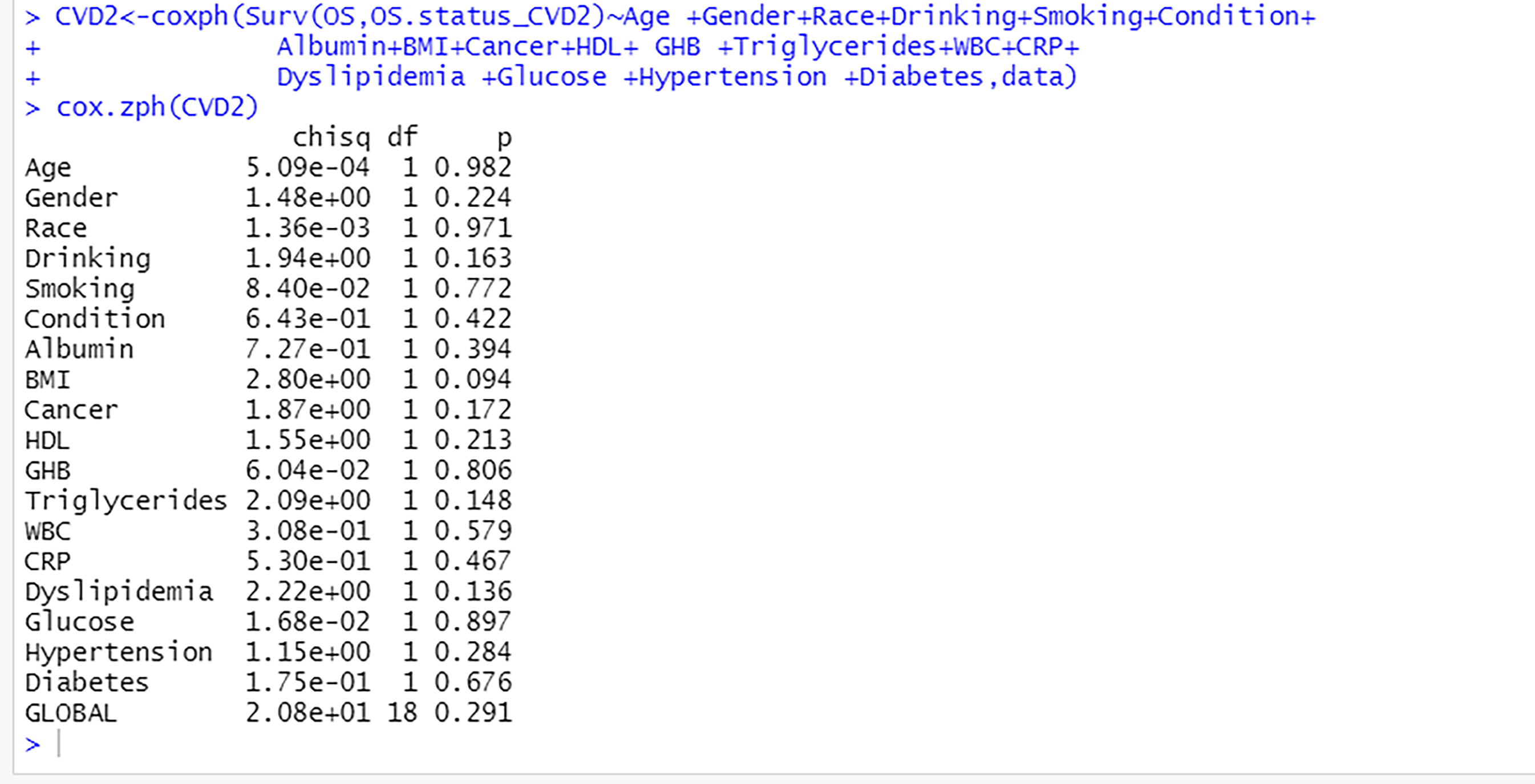

Supplement: Supplementary file 9 [file Image_8.TIF]
